# Transcranial direct current stimulation suggests a causal role of the medial prefrontal cortex in learning social hierarchy

**DOI:** 10.1038/s42003-024-05976-2

**Published:** 2024-03-09

**Authors:** Chen Qu, Yulong Huang, Rémi Philippe, Shenggang Cai, Edmund Derrington, Frédéric Moisan, Mengke Shi, Jean-Claude Dreher

**Affiliations:** 1 Center for Studies of Psychological Application, Guangdong Key Laboratory of Mental Health and Cognitive Science, South China Normal University, Guangzhou, China; 2 Department of Experimental Psychology, Ghent University, Ghent, Belgium; 3 Laboratory of Neuroeconomics, Institut des Sciences Cognitives Marc Jeannerod, CNRS, Lyon, France; 4 Université Claude Bernard Lyon 1, Lyon, France; 5 School of Economics and Management, South China Normal University, Guangzhou, China; 6 Key Lab for Behavioral Economic Science & Technology, South China Normal University, Guangzhou, China; 7 GATE CNRS and EmLyon, Ecully, France

**Keywords:** Learning algorithms, Decision, Forgetting

## Abstract

Social hierarchies can be inferred through observational learning of social relationships between individuals. Yet, little is known about the causal role of specific brain regions in learning hierarchies. Here, using transcranial direct current stimulation, we show a causal role of the medial prefrontal cortex (mPFC) in learning social versus non-social hierarchies. In a Training phase, participants acquired knowledge about social and non-social hierarchies by trial and error. During a Test phase, they were presented with two items from hierarchies that were never encountered together, requiring them to make transitive inferences. Anodal stimulation over mPFC impaired social compared with non-social hierarchy learning, and this modulation was influenced by the relative social rank of the members (higher or lower status). Anodal stimulation also impaired transitive inference making, but only during early blocks before learning was established. Together, these findings demonstrate a causal role of the mPFC in learning social ranks by observation.

## Abstract (web summary)

A brain stimulation study (tDCS) on healthy humans shows that the medial prefrontal cortex plays a causal role in learning social hierarchical relationships by observation.

## Introduction

We live in a social environment that is regulated by a variety of hierarchical structures^1^. Optimization of our social interactions requires us to perceive status cues and continuously update hierarchical relationships, by determining the power of others relative to ourselves, to make social judgments in daily life^2^. Social hierarchy exists in many species, including non-human primates^3^, rodents^4^, fish^5,6^, and humans^7,8^, which is crucial to maintaining the stability of populations and the health of individuals^2,9,10^. For example, animal studies have shown that burtoni fish can infer the social hierarchy of competitors by observation learning^6^, and clownfish can adjust their size and growth rate according to their hierarchical position in group^5^. Similarly, social hierarchy affects human behaviors^11^, such as decision-making^12^ and empathy^13^, enabling people to choose favorable alliances in social competition and avoid potential conflicts. Impairment in accurately monitoring one’s position in the social hierarchy can affect human health and increase the possibility of mental diseases such as social anxiety^10,14^.

Individuals can assess hierarchy information in several ways^2^, including via the perception of dominance-related cues (faces with dominant features, body postures, etc.), observational learning by trial and error^15,16^, and through competitive interactions^17^. Although assessing the strength of competitors by dominance cues, such as body postures, facial expressions, and physical attributes^18–20^ is rapid, the information from such cues does not always coincide with the real hierarchy status. In contrast, learning dominant relationships through direct dyadic competitive interactions, by experiencing successive victories or defeats against competitors, is time-consuming^17^, and may be costly in terms of potential physical injuries. Thus, learning social hierarchy by observation is an efficient way to acquire social hierarchy knowledge, without the cost incurred through competitive interactions.

Previous correlational fMRI studies indicated that learning social hierarchy engages the medial prefrontal cortex (mPFC), as well as the hippocampus^15–17^. Using model-based functional magnetic resonance imaging (fMRI), Kumaran et al.^15^ developed an observational hierarchy learning task that distinguished training and test phases to study the neural representations of the process of acquiring knowledge of hierarchies (during a training phase) and making transitive inferences on the basis of that knowledge (during a test phase). The mPFC was involved when learning the power information about other individuals in the social hierarchy^21^. In contrast, the hippocampus was involved in general hierarchy learning, i.e., the learning of both Social and Non-social hierarchies^16^. Recent models and experimental studies have proposed that the same brain representations that map space may be extended to a broad range of non-spatial problems in abstract cognitive space^22–26^. These studies support that the mPFC and the hippocampus are involved in non-spatial relational memory tasks allowing them to make transitive inferences^26–28^. Moreover, mPFC distinguishes between ranks higher and lower than oneself, and specifically shows reduced activity for trials involving higher social ranks^15^. The social comparison theory posits that people are driven to compare themselves with others for accurate self-evaluations^29^. Thus, people compare themselves to others in two opposite directions which may differ in motivation and comparison target, etc^30,31^. It remains unclear whether the mPFC, a key component of this mPFC-hippocampus network, is necessary for two distinct processes needed to organize abstract relational information into a cognitive map: acquiring knowledge of the relative rank between two adjacent items and making transitive inferences between items never presented together before, to guide novel inferences. It is also unknown whether mPFC perturbation affects knowledge acquisition and/or the making of transitive inference processes in similar ways across the social vs non-social domains. More specifically, mPFC perturbation may have distinct effects depending on whether the knowledge is pertinent to higher or lower social ranks.

Here, we aimed to examine the role of the mPFC in learning hierarchy relationship by observation using transcranial direct current stimulation (tDCS) approach. tDCS is a noninvasive brain stimulation method that modulates neural excitability of targeted brain regions using a low electrical current^32^. Our aims were thus: (i) to investigate whether the mPFC plays a key role when learning hierarchies or during the transitive inference processes; (ii) to establish whether the mPFC plays a specific role only for learning social, but not for non-social hierarchy and whether this is influenced by relative social rank (higher or lower status).

**Fig. 1:**
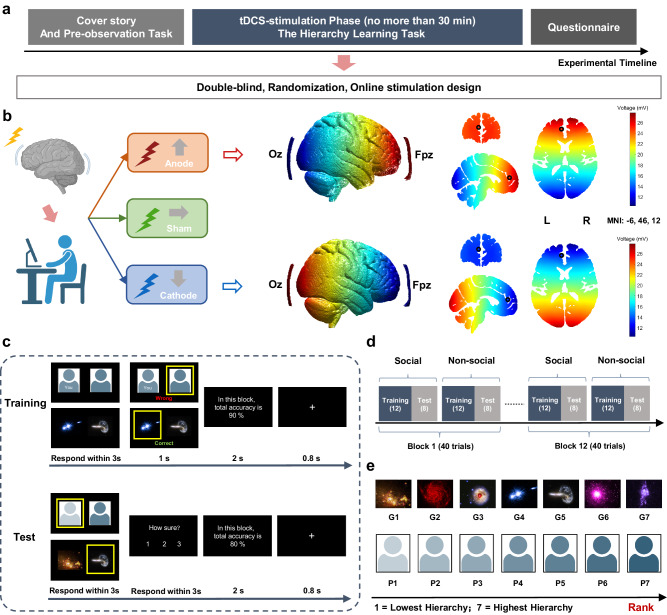
Illustration of the experimental procedure and behavioral paradigm. **a** Participants firstly were given a cover story. They were asked to imagine that they recently had joined a technology company that detected precious minerals in different galaxies. As new members of the company, they were instructed to learn the hierarchical relationships between the staff (Social) and the mineral contents of the different galaxies (Non-social). To familiarize themselves with the company members and galaxies, they were instructed to passively observe all the pictures (7 faces, 7 galaxies) in an pre-observation task. Each picture was randomly presented three times. Next, participants were randomly assigned to the anodal, sham, or cathodal stimulation, and instructed to perform a hierarchy learning task. At the end of the experiment, participants were required to complete questionnaires, including Social Dominance Orientation scale, and post-questions about the task and tDCS stimulation (see Methods for the details). **b** The Fpz-Oz montage of brain stimulation was chosen based on previous studies as targeting mPFC. Electric field simulation results for anodal and cathodal tDCS shown the simulated voltage distribution over the prefrontal cortex (left), and in coronal, sagittal, and axial slices (right) using the anodal montage with the MNI template brain. The black circle shows the targeted mPFC coordinates from Kumaran et al.^15^ (MNI: −6, 46, 12). The voltage indicates the strength of tDCS across the brain (L = left; R = right). **c** There are two phases in the hierarchy learning task. In the Training phase, participants were presented with adjacent items of the hierarchy (i.e., P4 vs P5, G4 vs G5, where P4 = “You”; and G4 = galaxy of rank equal to 4). They had to indicate the person they thought had higher status or the galaxy with more minerals. They received a feedback (correct/wrong) based on their choices allowing them to learn the hierarchical relationships between adjacent items. In the Test phase, participants were required to view non-adjacent items in the hierarchy (i.e., P1 vs P5, G1 vs G5), infer which one was the higher-ranked item, and to rate their confidence in their choice (no feedback was provided). **d** There are 12 blocks in the task including 12 training trials and 8 test trials in each block. The non-social condition was identical to the social condition except the stimuli were pictures of galaxies. **e** For the social condition, human faces were gender matched with real facial photos selected (here we use different color of silhouettes for illustration, see Methods for details). For the non-social condition, galaxy pictures were the same for females and males. The galaxy image courtesy of NASA and the Space Telescope Science Institute (STScI, http://hubblesite.org). (1=Lowest in hierarchy, 7=highest in hierarchy).

Using a double-blind sham-control, and online stimulation design, participants were randomly assigned to receive either anodal (*n* = 42), cathodal (*n* = 42), or sham (*n* = 44) stimulation over the mPFC (Fig. 1a). A stimulation montage Fpz-Oz with 1.5 mA current was adopted, using EEG10–20 system for electrode placement, across subjects (see Methods for more detail). As illustrated in Fig. 1b, the electric field simulation shows that the voltage gradient spread through the prefrontal cortex and targeted mPFC (MNI: −6, 46, 12; from Kumaran et al.^15^). During brain stimulation, participants performed a hierarchy learning task (Fig. 1c–e), including Training and Test phases for both social and non-social conditions. Self-information was added to the social condition to study how social ranks modulate the self-other comparison process of hierarchy learning. In the Training phase, participants were required to view pairs of hierarchically adjacent pictures and indicate which picture they thought had a higher status/power (Social) or more minerals (Non-social), with the correct feedback for each trial. Thus, through trial and error, they can update the hierarchical knowledge. In the Test phase, they were required to use the hierarchy information acquired during the Training phase to make transitive judgments concerning the hierarchical relationship between two non-adjacent entities (i.e., that were never seen together during training), with no feedback provided. They were also required to rate their confidence level from 1 (guess) to 3 (very sure), which allowed us to track the uncertainty of participants choices during the hierarchy learning. The construction of pairs in the testing phase was based on the approach used by Kumaran et al.^15,16^. It highlights the role of transitivity inference in social rank judgments, which is well-established using the transitive inference task with various dimensions such as length and weight in both human and animal research^6,33–35^. The construction of the two-phase hierarchy learning task allowed us to investigate hierarchy knowledge updating (Training) and transitive inferences (Test), respectively. Our findings show that the mPFC play a causal role in learning hierarchy and this involvement is more specific to the social conditions influenced by the relative social rank of the members. Anodal stimulation over mPFC impaired social hierarchy learning but also the early stage of social transitive inference.

## Results

To investigate how brain stimulation modulated hierarchy learning, we applied panel logistic or linear regression analyses, depending on the form of dependent variable (performance accuracy, reaction time, or confidence rating for each trial). We focused on the population-average effect using a generalized estimating equation (GEE) approach [see Methods for details of statistics]. These analyses were designed to capture the effect of stimulation over time at the level of the population. The independent variables were the tDCS stimulation (Anode/Sham/Cathode), hierarchy condition (Social/Non-social), and block number (1–12). We incorporated interaction terms to examine both two-way and three-way interactions. This approach allowed us to determine whether the effects of stimulation and hierarchy conditions varied at different stages of the experiment (as indicated by the block number) and to identify any potential synergistic effects among these variables. The percentage change effect was estimated via the average marginal effect (reported as *β* value below, see Methods). The marginal estimation calculates the average effect across all individuals and time periods in the panel dataset, which can easily be interpreted as the discreet change of the dependent variable given a unitary change of an independent variable.

### Effect of tDCS on learning hierarchical knowledge (training phase)

**Fig. 2:**
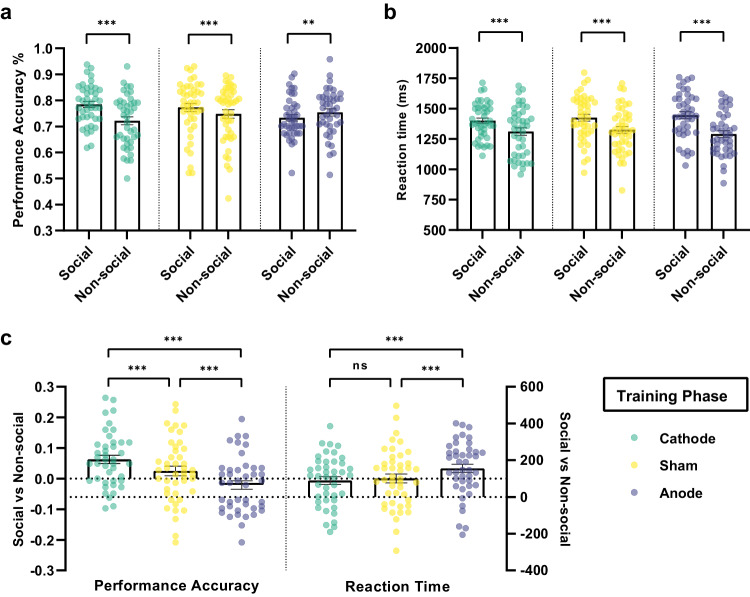
Effect of tDCS stimulation over mPFC on the Training Phase. **a** Performance accuracy (%) across blocks on social and non-social condition of different tDCS groups. **b** Reaction time (ms) across blocks on social and non-social condition of different tDCS groups. The y-axis of (**a**) and (**b**) indicate the raw performance data, with each dot representing an individual participant. The significance levels compare social and non-social conditions. **c** tDCS modulation of Social compared to Non-social hierarchy learning performance accuracy (left) and reaction time (right) across blocks during Training phase. The y-axis indicates the raw data of social minus non-social condition, with each dot representing an individual participant. The significance levels compare effects among Cathode, Sham, and Anode stimulation on the differences between social and non-social conditions. All significance levels labeled were estimated by the marginal average effect across all individuals and time periods in the panel regression (*N* = 128: Cathode=42, Sham=44, Anode=42; * indicates *p* < 0.05, ** indicates *p* < 0.005, *** indicates *p* < 0.001, ns indicates non-significant). Error bars indicate standard error of the mean.

We first focused on the impact of mPFC-targeted brain stimulation regimes on hierarchical learning behavior between the social and non-social conditions. During the training phase, the overall learning accuracy was 0.752 ± 0.080 *SD* (Sham = 0.760 ± 0.092 *SD*, Cathode = 0.753 ± 0.076 *SD*, Anode = 0.743 ± 0.069 *SD*). There was no significant difference in the overall performance among three tDCS groups when combining both social and non-social conditions. Panel logistic regression shows, under both cathodal and sham stimulation, participants learned better in the social condition relative to the non-social (Probability of accuracy Cathode Social > Non-social: *β* = 0.061, SE = 0.007, *z* = 8.27, *p* < 0.001, 95% CI (0.046, 0.075); Sham Social > Non-social: *β* = 0.024, SE = 0.070, *z* = 3.52, *p* < 0.001, 95% CI (0.011, 0.038); Fig. 2a). Moreover, in contrast to the non-social condition, cathodal stimulation increased social hierarchy learning compared to sham stimulation (Contrasts of average marginal effects Cathode > Sham in Training: *χ*^2^ (1) = 12.68, *p* < 0.001; Fig. 2c). On the contrary, anodal stimulation significantly decreased accuracy in the social condition compared to non-social (Anode Social < Non-social: *β* = −0.021, SE = 0.007, *z* = −2.80, *p* = 0.005, 95% CI (−0.035, −0.006); Contrasts of average marginal effects Anode < Sham in Training: *χ*^2^ (1) = 19.82, *p* < 0.0001; Fig. 2a, c). Furthermore, participants spent significantly more time to make decisions when facing the social hierarchy compared to non-social under anodal stimulation (Reaction Time Anode > Sham in Training: *χ*^2^(1) = 25.44, *p* < 0.0001; Fig. 2b, c), suggesting an impairment of updating/learning social hierarchy knowledge under anodal stimulation. In summary, brain stimulation over mPFC significantly influences the learning of hierarchical knowledge (Training) between the social and non-social conditions. Specifically, anodal stimulation tends to impair the learning process, whereas cathodal stimulation enhances the learning of social hierarchies compared to non-social contexts.

### Effect of tDCS on making hierarchical transitive inference (test phase)

During the test phase, the overall transitive inference accuracy was 0.806 ± 0.116 *SD* (Sham = 0.815 ± 0.119 *SD*, Cathode = 0.792 ± 0.132 *SD*, Anode = 0.812 ± 0.095 *SD*). There was no significant difference in the overall performance when combining both social and non-social conditions. To better understand the relationship between initial learning (Training phase) and subsequent test performance, we calculate the correlations between the overall performance of Training and Test phases for each stimulation condition. These revealed a positive correlation in all conditions (Sham: *r* _pearson_ = 0.709, *p* < 0.001, 95% CI (0.522, 0.831); Cathode: *r* _pearson_ = 0.559, *p* < 0.001, 95% CI (0.308, 0.738); Anode: *r* _pearson_ = 0.524, *p* < 0.001, 95% CI (0.262, 0.714)), with the strongest correlation in the sham condition. This pattern implicates that tDCS stimulation, in general, might disrupt the link between learning and inference performance.

**Fig. 3:**
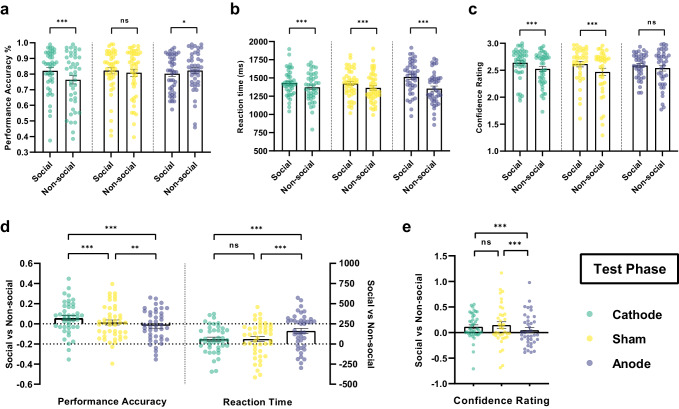
Effect of tDCS stimulation over mPFC on the Test Phase. **a** Performance accuracy (%) across blocks on social and non-social condition of different tDCS groups. **b** Reaction time (ms) across blocks on social and non-social condition of different tDCS groups. **c** Confidence rating on social and non-social condition of different tDCS groups. The y-axis of (**a**), (**b**), and (**c**) indicate the raw performance data, with each dot representing an individual participant. The significance levels compare social and non-social conditions. **d** tDCS modulation of Social compared to Non-social hierarchy learning performance accuracy (left) and reaction time (right) across blocks during Test phase. **e** tDCS modulation of Social compared to Non-social hierarchy learning confidence rating across blocks. The y-axis of (**d**) and (**e**) indicates the raw performance data of social minus non-social condition, with each dot representing an individual participant. The significance levels compare effects among Cathode, Sham, and Anode stimulation on the differences between social and non-social conditions. All significance levels labeled were estimated by the marginal average effect across all individuals and time periods in the panel regression (Accuracy and Reaction time *N* = 128: Cathode=42, Sham=44, Anode=42; Confidence rating *N* = 118: Cathode=41, Sham=38, Anode=39, see Methods for detailed; * indicates *p* < 0.05, ** indicates *p* < 0.005, *** indicates *p* < 0.001, ns indicates non-significant). Error bars indicate standard error of the mean.

As shown in Fig. 3a, d, under anodal stimulation participants’ performance to infer social hierarchy was significantly worse than on the non-social hierarchy task (Anode Social < Non-social: *β* = −0.020, SE = 0.008, *z* = −2.53, *p* < 0.05, 95% CI (−0.036, −0.005); Contrasts of average marginal effects Anode < Sham in Test: *χ*^2^(1) = 9.25, *p* < 0.005). In contrast, performance to infer social hierarchy was significantly better under cathodal stimulation (Cathode Social > Non-social: *β* = 0.055, SE = 0.008, *z* = 6.49, *p* < 0.001, 95% CI (0.038, 0.072); Contrasts of average marginal effects Cathode > Sham in Test: *χ*^2^(1) = 13.39, *p* < 0.0005). However, under sham stimulation, unlike during training trials, there was no difference in performance accuracy between social and non-social hierarchy learning when making transitive inferences (Sham: *β* = 0.013, SE = 0.008, *z* = 1.76, *p* = 0.078, 95% CI (−0.001, 0.028)). Similar to the Training phase, anodal stimulation significantly slowed the making of transitive inferences in social compared to non-social hierarchy (Reaction time Anode Social > Non-social: *β* = 159.783, SE = 9.861, *z* = 16.20, *p* < 0.0001, 95% CI (140.456, 179.109), Fig. 3b; Contrasts of average marginal effects Anode > Sham in Test: *χ*^2^ (1) = 52.2, *p* < 0.0001; Fig. 3d).

During Test trials, participants were also required to rate the confidence in their choices. We expected participants to show higher confidence rating in social hierarchy inference compared to the non-social condition. As shown in Fig. 3c, the analysis of confidence ratings in the transitive judgements showed that under cathodal and sham stimulation, in line with our hypothesis, participants were more confident in their social than non-social hierarchy decisions (Cathode Social > Non-social: *β* = 0.129, SE = 0.012, *z* = 10.6, *p* < 0.001, 95% CI (0.105, 0.153); Sham Social > Non-social: *β* = 0.155, SE = 0.013, *z* = 11.94, *p* < 0.001, 95% CI (0.130, 0.181)). This was not the case for the anodal stimulation group (Anode Social > Non-social: *β* = 0.022, SE = 0.127, *z* = 1.74, *p* = 0.081, 95% CI (−0.003, 0.047)). Moreover, in the social compared to non-social conditions, participants under anodal stimulation were less confident in their judgments compared to sham stimulation (Contrasts of average marginal effects Anode < Sham: *χ*^2^ (1) = 53.86, *p* < 0.0001; Fig. 3e]. These results are consistent with the worse performance observed under anodal tDCS in the social condition compared to the non-social, suggesting that participants were aware of their poorer performance in this condition.

Overall, the above findings indicate that brain stimulation over mPFC has different impacts between social and non-social hierarchy learning in both training and transitive inferences. Cathodal stimulation improved social hierarchy learning, while anodal stimulation impaired it. In the absence of tDCS (sham condition), participants showed a preference for learning social hierarchies over non-social, but there was no significant effect on transitive inferences.

### Anode stimulation impairs hierarchy learning in the social condition

**Fig. 4:**
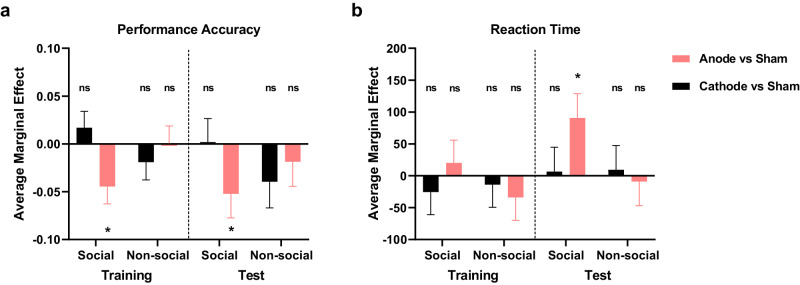
tDCS stimulation modulates social hierarchy learning on Training and Test phase. **a** Performance accuracy (%) across blocks on tDCS group compared to Sham condition (Anode vs Sham, and Cathode vs Sham) of social and non-social conditions during Training (left) and Test (right) phase. **b** Reaction time (ms) across blocks on tDCS group compared to Sham condition (Anode vs Sham, and Cathode vs Sham) of social and non-social conditions during Training (left) and Test (right) phase. The y-axis indicates the estimated average marginal effect. The significance labels indicate the comparison between Cathode and Sham, or Anode and Sham. All significance levels were estimated by the average marginal effect across all individuals and time periods in the panel dataset. All significance levels labeled were estimated by the marginal average effect across all individuals and time periods in the panel regression (* indicates *p* < 0.05, ** indicates *p* < 0.005, *** indicates *p* < 0.001, ns indicates non-significant). Error bars indicate standard error of the mean.

We estimated the marginal effect of learning a social or non-social hierarchy under each specific mPFC-targeted stimulation (Anode/Cathode) compared to Sham condition. This analysis was performed on the performance of Training and Test phases independently. In line with prior findings, anodal stimulation resulted in significantly lower accuracy during social hierarchy learning in both Training and Test trials, compared to Sham (Social Training Anode < Sham: *β* = −0.045, SE = 0.018, *z* = −2.45, *p* = 0.014, 95% CI (−0.08, −0.009); Social Test Anode < Sham: *β* = −0.052, SE = 0.025, *z* = −2.07, *p* = 0.038, 95% CI (−0.102, −0.003); Fig. 4a). On the other hand, we found cathodal stimulation improved the performance of social condition compared to non-social hierarchy learning. Unlike anodal, cathodal stimulation did not significantly influenced the social hierarchy learning (Social Training Cathode < Sham: *β* = 0.017, SE = 0.017, *z* = 0.99, *p* = 0.322, 95% CI (−0.017, 0.05); Social Test Cathode < Sham: *β* = 0.002, SE = 0.025, *z* = 0.07, *p* = 0.941, 95% CI (−0.047, 0.05)). Interestingly, neither anodal nor cathodal tDCS significantly influenced the learning of non-social hierarchies (Fig. 4a). This lack of effect highlights a potential specificity of the tDCS effects over mPFC on social hierarchy learning. Moreover, a notable effect of brain stimulation was observed on reaction time, but this was only evident during the test phase and specifically in the social condition (Social Test Anode > Sham: *β* = 90.619, SE = 38.061, *z* = 2.38, *p* = 0.017, 95% CI (16.021, 165.2161); Fig. 4b). Combined with the findings on learning performance, these results substantiate that tDCS stimulation over mPFC modulates social hierarchy learning, but not non-social hierarchy. Anodal stimulation appears to hinder the learning and transitive inference of social hierarchies. While cathodal stimulation does not exert a significant specific effect on social context, it results in a general improvement of learning social hierarchy knowledge compared to non-social.

### tDCS impact on block-to-block social hierarchical learning performance

**Fig. 5:**
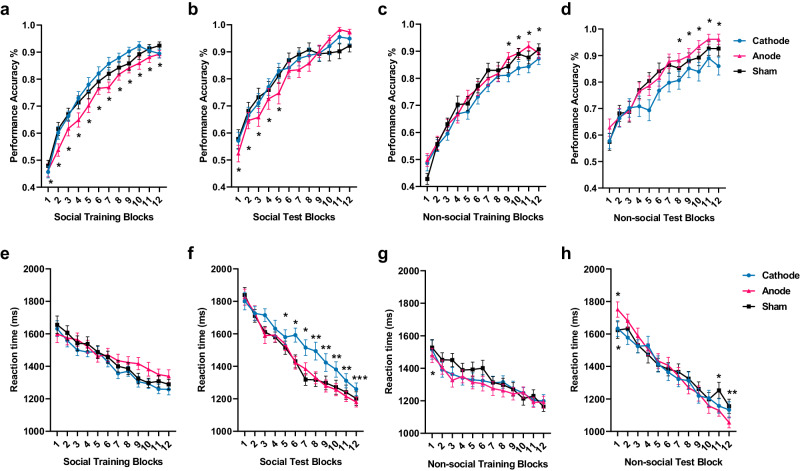
Effect of tDCS stimulation over mPFC on hierarchy learning from trial block to block. **a** Performance accuracy (%) modulated by tDCS from trial block to trial block over consecutive Social Training phase. **b** Performance accuracy (%) modulated by tDCS from trial block to trial block over consecutive Social Test phase. **c** Performance accuracy (%) modulated by tDCS from trial block to trial block over consecutive Non-social Training phase. **d** Performance accuracy (%) modulated by tDCS from trial block to trial block over consecutive Non-social Test phase. **e** Reaction time (ms) modulated by tDCS from trial block to trial block over consecutive Social Training phase. **f** Reaction time (ms) modulated by tDCS from trial block to trial block over consecutive Social Test phase. **g** Reaction time (ms) modulated by tDCS from trial block to trial block over consecutive Non-social Training phase. **h** Reaction time (ms) modulated by tDCS from trial block to trial block over consecutive Non-social Test phase. The y-axis from (**a**)–(**d**) indicates the raw performance data and the y-axis from (**e**)–(**h**) indicates reaction time (ms). The significance labels indicate the comparison between Cathode and Sham (on top of the lines), or Anode and Sham (on bottom of the lines). All significance levels were estimated by the average marginal effect across all individuals and time periods in the panel regression. Only significant results are labeled, see Supplementary Table 3−6 for the detailed significance slope comparison results of each block from the model estimation (*N* = 128: Cathode=42, Sham=44, Anode=42; * indicates *p* < 0.05, ** indicates *p* < 0.005, *** indicates *p* < 0.001). Error bars indicate standard error of the mean.

Next, we explored the effect of tDCS on the rate of learning hierarchical knowledge from trial block to trial block. We estimated the marginal effect of the block-to-block slope, in which the significance estimation reveals an increase or decrease of speed of learning (Fig. 5). There were contrasting effects of stimulation in the Training and Test phases.

First, there was a significant improvement in performance from block to block over consecutive blocks in both phases (Training block: *β* = 0.035, SE = 0.001, *z* = 54.88, *p* < 0.0001, 95% CI (0.035, 0.037); Test block: *β* = 0.031, SE = 0.001, *z* = 35.78, *p* < 0.0001, 95% CI (0.029, 0.032)). This effect confirms that participants were learning the hierarchies and were efficiently building on this learning to make successful transitive inferences as they progressed in the blocks. Note that the overall performance in the training phase is 0.752 ± 0.080 *SD* regardless of condition, while the performance in the test phase is 0.806 ± 0.116 *SD*. This difference between training and test performance was significant (*p* < 0.001), perhaps due to the design of the test trials. Indeed, for 6/7 of the elements, they either always win in every trial in which they are involved (i.e., P1, P2, and P3) or always lose (i.e., P5, P6, and P7). The only element that sometimes wins and sometimes loses was P4 (or G4 for non-social condition). This may have facilitated performance on the test trials. To test this, we conducted an analysis of test trials by separating those trials with and without P4/G4. We found the overall accuracy was significantly lower in the trials that involved P4/G4 compared to the trials without P4/G4 (*t* = −2.233, *p* = 0.027). This may explain why the performance of the test trials is higher than the performance of the learning trials, which included a higher proportion of trials containing ambiguous items that were sometimes lower status and sometimes higher depending on the item with which they were paired.

Second, there was no significant effect of tDCS on overall hierarchical learning performance during the trial and error Training blocks (same slopes in accuracy rate (block) estimated function; Cathode vs Sham: *χ*^2^(1) = 1.35, *p* = 0.24, Anode vs Sham: *χ*^2^(1) = 0.21, *p* = 0.65). However, there was a significant effect of anodal stimulation, which increased the rate of acquisition of the ability to make transitive inferences from the learned trial and error trials, when compared to sham tDCS, and cathode stimulation induced the opposite effect (Test block Slope Anode > Sham: *β* = 0.007, SE = 0.002, *χ*^2^(1) = 10.72, *p* = 0.0008, 95% CI (0.003, 0.011); Slope Cathode > Sham: *β* = −0.004, SE = 0.002, *χ*^2^(1) = 3.70, *p* = 0.046, 95% CI (−0.008, −0.00008)). These results show that mPFC stimulation did not affect the speed of learning adjacent stimuli (Training phase), but rather the use of previously acquired knowledge required to make transitive inferences of hierarchy.

Furthermore, when we investigated social and non-social learning separately, we found that anodal stimulation specifically impacted the accuracy of making transitive inferences in the social condition by improving the accuracy of performance from block to block (Test block Slope Anode > Sham: *β* = 0.009, SE = 0.003, *χ*^2^(1) = 11.82, *p* = 0.0006, 95% CI (0.004, 0.014); Slope Cathode > Sham: *β* = −0.004, SE = 0.003, *χ*^2^(1) = 2.74, *p* = 0.098, 95% CI (−0.010, 0.0008)), especially during earlier blocks (Fig. 5b and Supplementary Table 3, for Non-social see Fig. 5d and Supplementary Table 5). However, this effect appears to be the product of a generally worse performance during the training phase in each block, rather than any adverse effect on the making of transitive inferences per se. Indeed, the improved performance in making transitive inferences from block to block after anodal stimulation simply reflects that there is more room for improvement because of the lower performance during the preceding training trial blocks and the transitive inference blocks (Fig. 5a and Supplementary Table 3, for Non-social see Fig. 5c and Supplementary Table 5).

More intriguingly, in the social condition, anodal stimulation also resulted in a decrease in the rate of reduction of reaction times from trial block to trial block, during both the Training and Test phases, compared to sham, whereas cathodal stimulation resulted in no significant change (Social Training: Slope Anode > Sham: *β* = 9.580, SE = 2.207, *χ*2(1) = 18.84, *p* < 0.0001, 95% CI (5.254, 13.906); Slope Cathode > Sham: *β* = 0.285, SE = 2.207, *χ*^*2*^(1) = 0.02, *p* = 0.897, 95% CI (−4.041, 4.611), Fig. 5e; Social Test: Slope Anode > Sham: *β* = 9.970, SE = 2.824, *χ*^*2*^ (1) = 12.47, *p* = 0.0004, 95% CI (4.435, 15.505); Slope Cathode > Sham: *β* = 0.791, SE = 2.824, *χ*^*2*^ (1) = 0.08, *p* = 0.779, 95% CI (−4.743, 6.326), Fig. 5f; see Supplementary Table 4 for Social and Supplementary Table 6 and Fig. 5g, h for Non-social). This further suggests that anodal mPFC stimulation influences the modulation of social hierarchical knowledge updating and transitive inferences differently.

### Anode tDCS over mPFC impacts the learning of higher and lower social ranks differentially

**Fig. 6:**
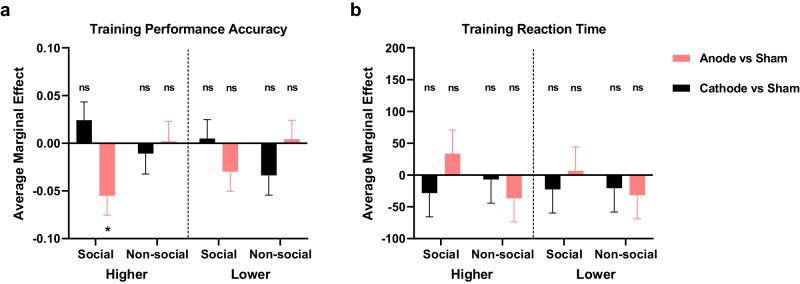
tDCS stimulation modulates social hierarchy learning basd on social ranks. **a** Performance accuracy (%) across blocks on tDCS group compared to Sham condition (Anode vs Sham, and Cathode vs Sham) of social and non-social conditions on higher ranks (left) and lower ranks (right) trials during Training phase. **b** Reaction time (ms) across blocks on tDCS group compared to Sham condition (Anode vs Sham, and Cathode vs Sham) of social and non-social conditions on higher ranks (left) and lower ranks (right) trials during Training phase. The y-axis indicates the estimated average marginal effect. The significance labels indicate the comparison between Cathode and Sham, or Anode and Sham. All significance levels were estimated by the average marginal effect across all individuals and time periods in the panel dataset. All significance levels labeled were estimated by the marginal average effect across all individuals and time periods in the panel regression (* indicates *p* < 0.05, ** indicates *p* < 0.005, *** indicates *p* < 0.001, ns indicates non-significant). Error bars indicate standard error of the mean.

Finally, to test whether the role of mPFC on learning social hierarchy is influenced by relative social rank (i.e., higher or lower status), we split trials according to those involving higher hierarchical status (Trials included Social: P4, P5, P6, P7; Non-social: G4, G5, G6, G7) and lower hierarchical status (Trials included Social: P1, P2, P3, P4; Non-social: G1, G2, G3, G4) in the Training phase. As shown in Fig. 6a, the tDCS-induced deficit in social hierarchy learning impinged asymmetrically on trials involving higher social ranks (Social higher ranks Anode < Sham: *β* = −0.055, SE = 0.020, *z* = −2.69, *p* = 0.007, 95% CI (−0.095, −0.015)), with no significant effect on trials involving lower social ranks (Social lower ranks Anode vs Sham: *β* = −0.030, SE = 0.021, *z* = −1.44, *p* = 0.150, 95% CI (−0.070, 0.011)). No similar asymmetrical effect was observed with respect to reaction times (Fig. 6b). This effect of anodal tCDS cannot be accounted for by self-involvement or related factors because it remained robust when we restricted our analysis to those trials that only involved the knowledge updating concerning other individuals in the hierarchy (i.e., trials that included the participants themselves were excluded (P4, and G4 in Non-social); Social training Accuracy Anode < Sham: *β* = −0.053, SE = 0.020, *z* = −2.69, *P* = 0.007, 95% CI (−0.091, −0.014); Social test Accuracy Anode < Sham: *β* = −0.047, SE = 0.026, *z* = −1.83, *P* = 0.068, 95% CI (−0.097, 0.003)). These results imply that the mPFC updated social hierarchy information specifically concerning members of the social hierarchy of higher status than oneself.

Moreover, as shown in Supplementary Table 2, there was no significant difference in age, choice bias, belief in cover story manipulation, sensation of tDCS stimulation, and social dominance orientation among the three tDCS stimulation groups. Thus, any effect of the groups on social hierarchy learning behavior cannot be accounted for by preexisting group differences or the sensation of the current stimulus. Last but not least, as shown in supplementary Fig. 1 and Supplementary Table 1 (see Supplementary Note 1 for details), the analysis of the first block trials in the Training phase did not found significant differences between groups in terms of initial performance. This additional analysis justify that the brain stimulation effect observed in our study was not due to the initial learning value differences within the tDCS groups.

Taken together, these results suggest a specific role of mPFC in tracking the development of knowledge about social, but not non-social hierarchies, and anodal tDCS results in an impairment on learning of social hierarchy knowledge and making of transitive inference judgments selectively focused on hierarchy members of higher status.

## Discussion

Our study examined the causal role of the mPFC in learning and making transitive inferences about social and non-social hierarchy relationships using tDCS stimulation. Anodal stimulation over the mPFC modulated social but not non-social hierarchy learning, which provides causal evidence implicating the mPFC in the establishment of social hierarchy knowledge. The two-phase hierarchy learning task adopted in our study allowed us to effectively separate the updating of hierarchical knowledge during the Training blocks from transitive inferences during the Test blocks. Anodal tDCS appeared to reduce the global performance of building and updating the social hierarchy knowledge during the Training phase. This also reduced the success of transitive inference making during the Test phases in the anodal group, because the learning of the social ranks was less well established during the early blocks of Training phase. However, during later blocks of the test phase, when the learning of the social hierarchy improved, the detrimental effect of anodal tDCS gradually decreased and disappeared (Fig. 5b). This shows that anodal tDCS does not disturb the making of inferences for social hierarchy once the learning of social ranks had improved. Thus, the apparent reduced accuracy observed in the early blocks of the test phase in the anodal group, for social vs non-social hierarchies, is likely a result of impaired learning of the social hierarchy in the early stages of training (Fig. 5a). Therefore, it appears that anodal stimulation of the mPFC disrupts the learning of social hierarchies, rather than the ability to make transitive inferences from them.

Our findings that anodal mPFC stimulation disrupts the learning of social hierarchies, but leaves intact the learning of non-social hierarchies, indicate that the learning of these two types of hierarchies may rely on distinct cognitive processes. The fact that social hierarchy depends on the mPFC resonates with recent studies suggesting that task representations may differ across domains, such as the spatial and conceptual domains^36^, or abstract vs naturalistic domains^37^. Here, the nature of the items themselves (faces vs galaxies) may have influenced how they were learned, because faces may be learned more easily. Confirming this hypothesis, differences in both learning accuracy and confidence were observed when directly comparing the sham group in the Social and Non-social conditions. Moreover, our experimental design allowed us to isolate the learning and representation of social and non-social information from prior knowledge in two ways. First, the power of individuals/items in both social and non-social hierarchies had to be learned without prior knowledge, making previously known information about oneself irrelevant. Second, it could be argued that the non-social task is not an ideal comparison to the social task, in that there is no self-relevance comparison of the non-social task. Indeed, the structure of the social and non-social tasks are identical, but G4 is not related to self while participants have a reference point (being themselves as P4) in the social task. To rule out this potential concern, additional analyses in which we excluded both trials that involved the participants themselves (referred to as “you” trials, i.e., trials involved P4), and non-social “G4” trials, confirmed that the effects we observed in the mPFC-targeted stimulation were specifically related to the social condition. Thus, our results provide strong evidence that the mPFC plays a causal role in the neural mechanisms for processing information related to others. This has important implications for understanding the causal role of this brain region in learning self-other relatonships^21^.

Another important finding from our study is that mPFC stimulation left the transitive inference-making processes unimpaired. This indicates that the mPFC is not specifically necessary to make transitive inferences. A recent simulation of electrode fields has shown that our montage of Fpz-Oz generates higher electric currents in the amygdala and the hippocampus, which might facilitate the modulation of deep brain regions^38^. The impact of the mPFC anodal stimulation on learning social hierarchy behavior may not be mediated by local activity alone, but by directed communication with other brain areas. For example, the hippocampus has been reported to encode abstract general knowledge of relationships whatever their nature (i.e. spatial, social, and non-social)^39^. The hippocampus is not only involved in forming cognitive maps to organize information simultaneously^28,40^, it also contributes to concept learning by representing the feature combinations related to current behaviors^41,42^. The mPFC represents stimuli-outcome relationships of cognitive maps^43^ and receives input information from the hippocampus to update current information^44^. Consistent with this, the mPFC was found to selectively mediate the learning of knowledge about social hierarchy, whereas domain-general coding of ranks was observed in the hippocampus, even when the task did not require it^15^. Another fMRI report did not identify the mPFC in social vs non-social hierarchy learning^16^, but the correlational nature of this fMRI study cannot account for a causal role of the mPFC in such learning process. Functional coupling between the mPFC and hippocampus has been shown to support social learning and is also involved in both conceptual learning and episodic memory of cognitive maps^45,46^. This may explain why mPFC-targeted tDCS stimulation perturbed the social hierarchy knowledge updating across blocks, but only impaired the transitive inference during the earlier blocks when the conceptual knowledge was not yet well established.

Our results show that anodal tDCS perturbs performance in an asymmetrical manner, and preferentially impinges on social comparison processes concerning those of status superior to one’s self, but having no significant effect on those ranked below. The social comparison theory posits that people are driven to compare themselves with others for accurate self-evaluations^29^. Specifically, people compare themselves with others in two opposite directions, downward and upward, that differ in motivations, comparison targets, and consequences^30,31^. The upward comparison refers to comparing with those who are thought to be ranked higher. Upward comparison is most likely performed to fulfill the motivation of challenging others and self-improvement. This type of social comparison invokes threat to the self^47^ and provokes negative emotions such as envy^48,49^. In contrast, the downward comparison is most likely performed to fulfill the motivation of self-enhancement. A previous study also suggests the causal role of dmPFC in self-other mergence^50^. Our findings that the mPFC is engaged or focused on individuals that rank higher than oneself demonstrate that this region is causally necessary for upward comparison. Our results agree with fMRI findings reporting that mPFC distinguishes between higher and lower ranks with respect to oneself^15^, and engagement in different types of social valuation processes^51,52^.

Last but not least, while anodal tDCS over the mPFC was found to selectively hinder social hierarchy learning performance, cathodal stimulation did not produce a selective corresponding enhancement. There are two primary hypotheses for this absence of a cathodal effect. Firstly, previous meta-analysis shown the effects of cathodal tDCS on behavior tend to be less stable and less likely to inhibit neural activity in comparison to anodal stimulation^53^. Secondly, the high baseline performance observed in the sham condition in our task implies that there was limited scope for further improvement in hierarchy learning through tDCS. In this light, exploring the impact of cathodal mPFC tDCS in individuals with specific deficits in social hierarchy learning might be insightful. Importantly, the lack of enhancement from cathodal stimulation indicates that the learning disruptions caused by anodal tDCS are attributable to its specific stimulation effects rather than general tDCS side effects (i.e. discomfort or distraction). This is supported by the fact that a reversal of stimulation polarity^54^ did not lead to a corresponding decrease in learning performance.

Overall, our tDCS approach establishes a causal relationship between mPFC and social hierarchy learning. The maladaptive assessment of social dominance hierarchies is an important source of distress in social disorders such as depression and anxiety^10,55^. Our findings not only extend our understanding of the role of mPFC and its involvement in social learning processes but also enriched our knowledge of brain stimulation techniques as a potential treatment for neuropsychiatric disorders (e.g., depression, anxiety) in which the experience of repeated social defeats often leads to social avoidance^2,56^.

## Methods

### Participants

A total of 136 participants (67 males, 69 females) were recruited via online fliers with informed written consent. All participants were right-handed, with no history of psychiatric or neurologic disorders, and were randomly assigned to receive anode, cathode, or sham stimulation over the medial prefrontal cortex (mPFC) while performing the hierarchy learning tasks. We set a threshold of 80% accuracy in Training phase. Six participants did not reach this priori threshold and were excluded from the analysis because they did not learn either Social or Non-social conditions in the training trials sufficiently well (the accuracy rate of each block was lower than 2/3). In addition, one participant was excluded because he responded stereotypically (i.e., one key for the whole block), and another because the program was restarted twice. Thus, the data from 128 participants (males=63, mean age=19.90 ± 0.145) were analyzed (Sham=44: Male=23, Female=21; Cathode=42: Male=21, Female=21; Anode=42: Male=21, Female=21). During the analysis of confidence ratings in the test phase, 9 participants were further excluded due to operational issues, which the program did not record their responses. 1 participant was excluded from the analysis because she consistently chose the lowest rating, “1,” even though she had a 100% accuracy rate. After the exclusion, the data of 118 participants were maintained for the confidence rating analysis (Anode=39, Sham=38, Cathode=41). The study was approved by the ethics committee of South China Normal University and all participants received 45 CNY after the task.

### Stimuli

Images of faces in the social condition were selected from the CAS-PEAL Large-Scale Chinese Face Database^57^. Silhouettes (2 faces, 1 female, 1 male), were used to represent “You” which refers to the participant in the experiment. Frontal images (12 neutral faces, 6 females, 6 males) identified fictive hierarchy members for the subsequent experiments. The hair and neck were preserved for the facial pictures. For female participants, the hierarchy was composed of pictures of females and vice versa for male participants. Previous studies have shown facial gender can influence perceptions, judgments, and behavior related to social hierarchy and social dominance^58–60^. By matching the gender between participants and stimuli we sought to ensure that participants perceived and evaluated the facial stimuli consistent with their own gender-related expectations, to reduce the number of potential factors that might influence the effect of social hierarchy learning and tDCS stimulation. Images of galaxies were selected from a public astronomy website (http://hubblesite.org). All pictures were processed by Adobe Photoshop software to ensure grayscale and resolution were consistent. The hierarchical ranks of galaxies and face stimuli were randomized across the groups. The experiment was programmed in E-prime 2.0 and presented on a 14-inch laptop.

### Experimental procedure

With a double-blind and sham-control design, our study included three phases: Cover story and Pre-observation Task, tDCS stimulation phase (Hierarchical Learning Task), and Questionnaires (Fig. 1a).

### Cover story

Participants were asked to imagine they had joined recently a technology company that detected precious minerals in different galaxies. Then, they were instructed to observe the photos of staff members and galaxies related to the company to familiarize themselves with company members and business (see Pre-observation task below). After the observation task, they were informed that as a new member of the company, they needed to learn the rank relationship between staff members to help them adjust to work. In the meantime, they also needed to learn the relative mineral contents of different galaxies. The learning task included two phases. During a training phase, within each trial, they will see a pair of pictures (i.e., faces or galaxies), they will need to choose which one has more power or minerals, and following the decision they will have feedback of their choice. During a test phase, within each trial, they will again see a pair of pictures, however, there will be no feedback of their choice provided and they need to rate their confidence in the choice from 1 to 3. At the end of each block, they have feedback on the accuracy rate of the current block. They were informed that the paired items in the training and test phase are different, their task is to learn as much as they can in both training and test phases.

### Pre-observation task

The pre-observation task was to reduce the differential effects of extraneous stimuli during subsequent tasks. Participants were instructed to passively observe the pictures presented on the screen to familiarize themselves with the staff members in the company and the galaxies related to the company business. There was a silhouette that represents “You” which referred to the participant in the experiment. There were three blocks of the task. Each block included 14 trials (7 face pictures, 7 galaxy pictures; randomly presented). Within each trial, following an 800 ms attention cross, the picture was presented for 3 s on the screen. Thus, participants observed each picture three times.

### Hierarchy learning task (performed under sham or tDCS stimulation)

During tDCS stimulation, participants were required to perform a hierarchy learning task, including Training and Test phases in both Social and Non-Social conditions. The condition presented first was consistent with the observation task and balanced pseudo-randomly among the participants. The sequences of paired pictures were randomized, as was the left or right location in which pictures were presented. Human faces (sex matched) were used in the Social condition, whereas images of galaxies were used in Non-Social condition.

In the Training Phase, participants were required to view a pair of adjacent hierarchical pictures (P4 vs P5, G4 vs G5; P=person and G=galaxy; P4 means “YOU”; Fig. 1c) and identify which picture they thought had a higher rank (Social) or more minerals (Non-Social). They received accurate feedback of the correctness of their selection, and were thus able to learn the hierarchical relationships between the adjacent items. Based on the studies of Kumaran et al.^15,16^, in the non-social condition, we omitted self-relevance information to focus solely on the cognitive mechanisms of hierarchical processing without personal associations. This approach provided a stark contrast to the social condition, where self-relevance is naturally embedded. There were 12 blocks of Training phase, with each block including 2 six trial mini-blocks composed of the 6 paired items (P1 vs P2, P2 vs P3, P3 vs P4, P4 vs P5, P5 vs P6, P6 vs P7). Each Training trial block was followed by a Test trial block.

For the Test Phase blocks, hierarchically non-adjacent pairs of pictures were presented (i.e., P1 vs P5, G1 vs G5; Fig. 1c). Participants were required to make transitive inference judgments and rate the confidence of their decisions from 1 (guess) to 3 (very sure). There were 12 blocks of Test phase and each included a single 8-trial mini-block composed of 8 paired items (P1 vs P4, P2 vs P4, P2 vs P5, P2 vs P6, P3 vs P5, P3 vs P6, P4 vs P6, P1 vs P7). Notably, we acknowledge that more challenging pairs in the test phase, such as P1 vs P3 and P5 vs P7, could provide valuable insights. However, we did not include these pairs because they involve only higher-than-self (P4) items (i.e., P5, P7), or only lower-than-self items (i.e., P1, P3), which might involve hierarchical preferences in transitive inference. The aim of the test phase was to evaluate the understanding of the hierarchy in relation to self-relevance.

For both Training and Test trials, at the end of each block, they received average accuracy feedback on their decisions.

### Questionnaires

After the Hierarchy learning task, participants were required to fill in the Social Dominance Orientation (SDO) scale, and answer the post-questions about the task and tDCS stimulation rating: i) the discomfort of electrode stimulation (from 1 for none to 5 for very discomforting); ii) how much they believed that they were one of the members of the company (from 1 for none to 10 for complete belief). A previous study found that the sensitivity of the right DLPFC to social ranks correlated positively with the SDO scale^61^, which is known to predict behaviors and political attitudes associated with the legitimization of dominance hierarchies^62^. Thus, the purpose of the SDO measure was to control for any group difference in sensitivity to social rank.

### Brain stimulation and current modeling

We used NeuroConn transcranial direct current stimulation devices (NeuroConn, Ilmenau, Germany) for tDCS stimulation. According to previous studies which investigated the causal role of mPFC in social behaviors^63,64^, we adopted the Fpz-Oz montage (EEG 10–20 system) with 1.5 mA current as the stimulation protocol. The Fpz-Oz montage has been shown in a recent simulation of electrode fields study that not only targeted the mPFC regions but also generates higher electric currents in the amygdala and the hippocampus, which might facilitate the modulation of deep brain regions^38^. We used gel to improve conductivity and reduce skin irritation. The electrode sizes were both 5 cm × 7 cm (35 cm^2^) (Fig. 1b). In all stimulation conditions, the current intensity was 1.5 mA, applied with a 30-second fade-in and fade-out at the beginning, and the end of the stimulation. For both anode and cathode stimulation, the 1.5 mA stimulus lasted no more than 30 min (when participants completed the task in less than 30 min, the current was terminated earlier). For sham stimulation, the current only lasted 15 s. To account for possible delays in the onset of tDCS effects, participants were required to wait 2.5 min after the onset of stimulation to start the hierarchy learning task (thus, for the sham condition, the current stimulation had ceased 2.25 min before the first learning trial).

To ensure that our electrode montage effectively stimulated the mPFC, electrical potential simulations were performed using ROAST^65,66^ with the MNI152 template brain. Electrodes were simulated as pads, with a 70x50x3mm pad located over Fpz and Cz of standard 10-10 system locations. Tissue conductivities were set as white matter=0.11 S/m, gray matter=0.21 S/m, CSF = 0.53 S/m, bone=0.02 S/m, and skin=0.90 S/m. For the anodal stimulation, 1.5 mA was set as inward flowing current from the Fpz, and −1.5 mA outward flowing current from the Cz. For the cathodal stimulation this was reversed.

### Statistics and reproducibility

Behavioral analyses were conducted in STATA 14. We employed a panel regression model to examine the impact of various predictors on the likelihood of (performance accuracy/reaction times/confidence rating). This analysis was conducted using function “xtlogit” or “xtreg” in STATA 14. For the panel regressions using the population-average effect (generalized estimating equation approach, GEE), this allowed us to estimate the effect of brain stimulation at the level of population and take into account the effect of time (Learning). The independent variables were the tDCS stimulation (Anode/Sham/Cathode), hierarchy condition (Social/Non-social), and block number (1–12). We incorporated interaction terms to examine both two-way and three-way interactions.

The Panel data of *t* = 480 trials clustered on each of *i* = 128 participants were used. We reported significant marginal effect of estimated β values. The model equation of GEE for panel logistic regressions:

$${{{{{\rm{logit}}}}}}(P({{{{{{\rm{Y}}}}}}}_{{{{{{\rm{it}}}}}}}=1))={{{{{{\rm{X}}}}}}}_{{{{{{\rm{it}}}}}}}* {{{{{\rm{\beta }}}}}}$$

*Y*_it_ represents the binary dependent variable for individual *i* at time *t*. *X*_it_ represents the vector of explanatory variables for individual *i* at time *t*. *P* represents the probability of a given event. β is the vector of coefficients to be estimated, representing the population-average marginal effects.

The marginal effect of the variable *X*_it_ is estimated as:

$${{{{{\rm{\beta }}}}}}=\sum [{{{{{\rm{P}}}}}}({{{{{{\rm{Y}}}}}}}_{{{{{{\rm{it}}}}}}}=1)* (1-P({{{{{{\rm{Y}}}}}}}_{{{{{{\rm{it}}}}}}}=1))* {{{{{{\rm{X}}}}}}}_{{{{{{\rm{it}}}}}}}* {{{{{{\rm{\beta }}}}}}}_{{{{{{\rm{j}}}}}}}]/{{{{{\rm{N}}}}}}$$

β represents the estimated marginal effect. *P*(Y_it_ = 1) represents the predicted probability of the dependent variable being equal to 1 for individual *i* at time *t*. *X*_it_ represents the vector of explanatory variables for individual *i* at time *t*. β_j_ represents the estimated coefficients from the GEE model. *N* represents the total number of observations in the panel dataset.

The marginal effect estimation equation calculates the average marginal effect across all individuals and time periods in the panel dataset. Thus, the estimated marginal effect can easily be interpreted as the discreet change of the dependent variable given a unitary change of an independent variable. It quantifies the change in the log-odds of the dependent variable associated with a one-unit change in the corresponding independent variable, while holding all other variables constant. In other words, it quantifies the impact of a unit change in the independent variable on the dependent variable, taking into account the effects of other variables in the model.

For the panel linear regressions, reported β value represents *X*_it_, i.e. the regression coefficient. Indeed, in a linear regression, the marginal effect of a variable is equal to the estimated coefficient.

### Reporting summary

Further information on research design is available in the Nature Portfolio Reporting Summary linked to this article.

### Supplementary information

Supplementary Information

Description of Additional Supplementary Files

Supplementary Data 1

Reporting Summary

## Data Availability

The behavioral data that support the findings of this study are available on Zenodo with the identifier: 10.5281/zenodo.10650865^67^. The source data behind the graphs in the paper can be found in Supplementary Data 1.

## Code availability

Software packages used for the analyses are STATA 14. All code used to generate the analyses is available on Zenodo with the identifier: 10.5281/zenodo.10650865^67^.
