# Supplementary material for: Transcranial direct current stimulation suggests a causal role of the medial prefrontal cortex in learning social hierarchy
Source: Commun Biol. 2024 Mar 9;7:304. doi: 10.1038/s42003-024-05976-2 (PMC10924847; doi:10.1038/s42003-024-05976-2)
Supplement: Supplementary file 1 — Supplementary Information [file 42003_2024_5976_MOESM1_ESM.pdf]

Supplementary Information for

Transcranial Direct Current Stimulation Suggests a Causal Role of the Medial Prefrontal Cortex in Learning Social Hierarchy

Chen Qu[1]*, Yulong Huang[1,2]*, Rémi Philippe[3,4]*, Shenggang Cai[5,6], Edmund Derrington[3,4], Frédéric Moisan[7], Mengke Shi [1], and Jean-Claude Dreher[3,4]

[1] Center for Studies of Psychological Application, South China Normal University, Guangzhou, China
[2] Department of Experimental Psychology, Ghent University, Ghent, Belgium
[3] Laboratory of Neuroeconomics, Institut des Sciences Cognitives Marc Jeannerod, CNRS, Lyon, France
[4] Université Claude Bernard Lyon 1, Lyon, France
[5] School of Economics and Management, South China Normal University, Guangzhou, China
[6] Key Lab for Behavioral Economic Science & Technology, South China Normal University, Guangzhou, China
[7] GATE CNRS and EmLyon, Ecully, France

* These authors contributed equally
Corresponding: Jean-Claude Dreher
Email: dreher@isc.cnrs.fr

This Supplementary Information includes:

Supplementary Note 1

Supplementary Figure 1

Supplementary Table 1 to 6

Supplementary Note 1

Supplementary analysis of the first block learning effect.

To justify that the brain stimulation effect was not due to the initial learning value differences within the group, we performed an additional analysis of the first block of the training phase. Note that within each training block participant repeated the learning of adjacent items twice. Thus, the average accuracy of the first block already included the learning effect through trial and error. To address the question of whether there is a different initial value between groups, we conducted two analyses: 1) Repeated measure ANOVA with Hierarchy Type (Social, Non-social) x tDCS (Cathode, Sham, Anode) x Familiarity (First-time trials, Second-time trials) on the first block performance accuracy (%) and reaction time (ms); 2) One-sample Student t-test of performance accuracy of each condition to test of the average accuracy is significantly greater than 0.5 chance level.

As shown in Supplementary Fig.1 a-b, the results of accuracy showed only a significant main effect of familiarity between the first-time and second-time trials ($F_{(1,125)} = 68.956$, $p < 0.001$, $\eta_p^2 = 0.356$; First-time trials: $M = 0.398$, $SD = 0.196$; Second-time trials: $M = 0.538$, $SD = 0.204$), indicating the participants in all the groups performed better in the second-time trials compared to the first time, and thus there was a learning effect within the first block. However, there was no significant three-way interaction ($F_{(2,125)} = 1.014$, $p = 0.366$, $\eta_p^2 = 0.016$) or two-way interaction between either hierarchy type and tDCS groups ($F_{(2,125)} = 1.014$, $p = 0.366$, $\eta_p^2 = 0.016$), or Familiarity and tDCS groups ($F_{(2,125)} = 2.606$, $p = 0.078$, $\eta_p^2 = 0.040$). These results indicate that for first-time and second-time trials, we did not observe differences between groups but only a general increased accuracy for the second-time trials within the first block. This provides evidence that there was no significant difference in the initial level of performance between the groups.

The analysis of reaction time showed similar results (see Supplementary Fig.1 c-d), i.e., within groups participants responded significantly slower during second-time trials compared to the first-time ($F_{(1,125)} = 68.956$, $p < 0.05$, $\eta_p^2 = 0.046$; First-time trials: $M = 1501.073$, $SD = 356.07$; Second-time trials: $M = 1545.291$, $SD = 337.691$). They also responded faster in the non-social condition compared to the social hierarchy training ($F_{(1,125)} = 15.978$, $p < 0.001$, $\eta_p^2 = 0.113$; Non-social: $M = 1471.743$, $SD = 348.474$; Social: $M = 1574.621$, $SD = 339.199$). However, there was no significant three-way interaction ($F_{(2,125)} = 0.589$, $p = 0.556$, $\eta_p^2 = 0.009$) or two-way interaction in either hierarchy type and tDCS groups ($F_{(2,125)} = 0.089$, $p = 0.915$, $\eta_p^2 = 0.001$), or Familiarity and tDCS groups ($F_{(2,125)} = 1.223$, $p = 0.298$, $\eta_p^2 = 0.019$). These results further confirm that at the beginning of the task, there was no group difference but only a general learning effect such that on the second-time trials, after the feedback from first-time trials, participants spent more time making a judgment and, compared to the non-social hierarchy, they took more time to make these judgments in the

social condition. Finally, a second one-sample student t-test (>0.5 chance level, see Supplementary Table 1) showed that only the Anode groups had significantly higher chance levels of accuracy on the second-time trials of the non-social hierarchy training. The other groups (cathode and sham) and all groups for the social condition were not significantly better than the chance level, indicating that participants were still at the initial phase of learning the hierarchies.

Supplementary Figure 1: First block learning effect of the Training phase.

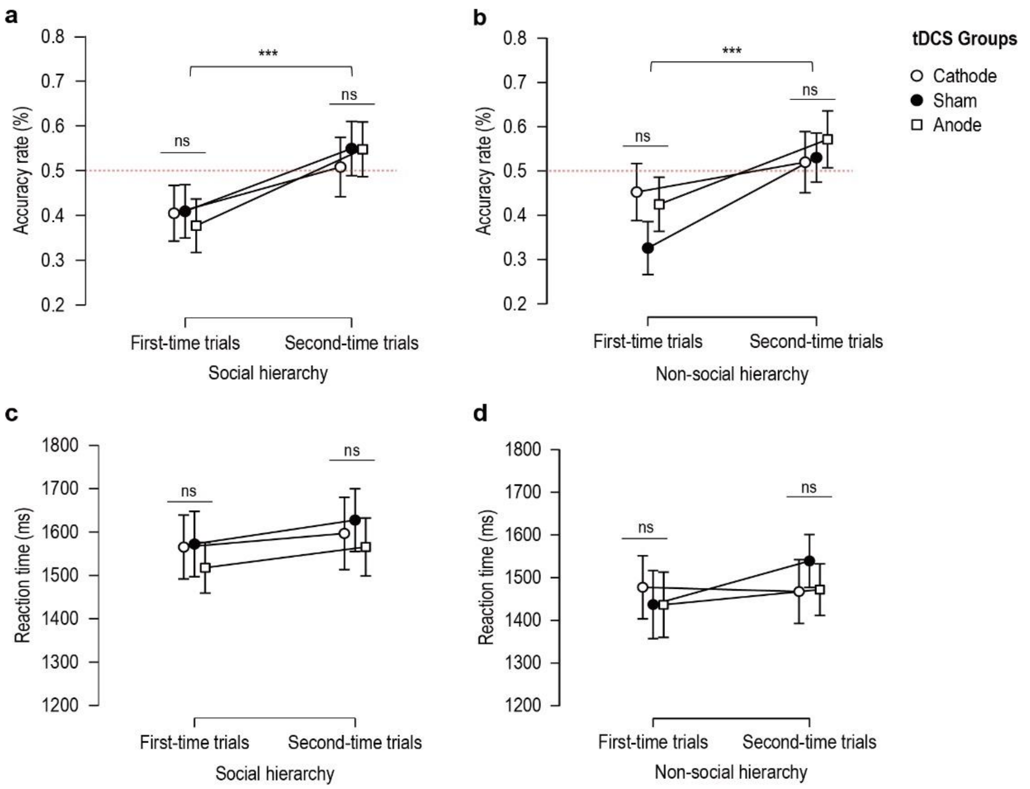

a Performance accuracy (%) of first block trials on Social Training Phase in different tDCS groups. **b** Performance accuracy (%) of first block trials on Non-social Training Phase in different tDCS groups. **c** Reaction time (ms) of first block trials on Social Training Phase in different tDCS groups. **d** Reaction time (ms) of first block trials on Non-social Training Phase in different tDCS groups. The y-axis indicates the raw performance data. (N=128: Cathode=42, Sham=44, Anode=42; * indicates $p < 0.05$, ** indicates $p < 0.005$, *** indicates $p < 0.001$, ns indicates non-significant). Error bars indicate Confidence Intervals 95% CI.

Supplementary Table 1

								95% CI for Cohen's d	
**Hierarchy type**	**Familiarity**	**tDCS**	**Mean**	**SD**	**t**	**p**	**Cohen's d**	**Lower**	**Upper**
Non-social	First-time	Anode	0.425	0.195	-2.286	0.986	-0.357	-0.62	∞
		Cathode	0.452	0.231	-1.338	0.906	-0.209	-0.467	∞
		Sham	0.326	0.183	-6.312	1	-0.952	-1.247	∞
	Second-time	Anode	0.571	0.202	2.293	0.014*	0.358	0.091	∞
		Cathode	0.52	0.233	0.666	0.255	0.104	-0.154	∞
		Sham	0.53	0.184	1.091	0.141	0.164	-0.086	∞
Social	First-time	Anode	0.377	0.188	-4.107	1	-0.641	-0.92	∞
		Cathode	0.405	0.188	-3.147	0.998	-0.492	-0.761	∞
		Sham	0.409	0.174	-3.464	0.999	-0.522	-0.784	∞
	Second-time	Anode	0.548	0.206	1.379	0.088	0.215	-0.046	∞
		Cathode	0.508	0.198	0.26	0.398	0.041	-0.217	∞
		Sham	0.549	0.205	1.592	0.059	0.24	-0.013	∞

Note. For the Student t-test, the alternative hypothesis specifies that the mean is greater than 0.5. *<0.05

Supplementary Table 2. Descriptive statistics of Demographic, Questionnaires, and Task-relevant subjective rating

		Anodal (N=42)	*Sham (N=44)*	*Cathodal (N=42)*
Gender		Males=21<br>Females=21	Males=22<br>Females=23	Males=21<br>Females=21
Age		19.610±0.231	19.909±0.239	20.171±0.281
SDO		51.190±13.515	53.114±12.085	51.595±10.378
Truth Degree		5.452±0.350	5.545±0.337	5.976±0.352
tDCS uncomfortable rating		1.536±0.146	1.341±0.101	1.762±0.137
Choice Bias	**Training**	Left=0.501±0.005<br>Right=0.499±0.005	Left=0.504±0.005<br>Right=0.496±0.005	Left=0.503±0.005<br>Right=0.497±0.005
	**Test**	Left=0.502±0.003<br>Right=0.498±0.003	Left=0.508±0.003<br>Right=0.492±0.003	Left=0.499±0.003<br>Right=0.501±0.003

Note: One-way ANOVA showed no significant differences of the above measurements among groups.

Supplementary Table 3. tDCS effect of block-to-block hierarchy learning in the performance % of Social condition

	Anode vs Sham				*Cathode vs Sham*			
	Training		Test		Training		Test	
Block	**b(SE)**	**P**	**b(SE)**	**P**	**b(SE)**	**P**	**b(SE)**	**P**
1	-0.053(0.026)	0.044*	-0.113(0.036)	0.002**	0.002(0.026)	0.925	0.037(0.036)	0.305
2	-0.055(0.025)	0.028*	-0.103(0.035)	0.004**	0.009(0.025)	0.728	0.026(0.035)	0.451
3	-0.055(0.024)	0.020*	-0.090(0.035)	0.009**	0.014(0.023)	0.554	0.016(0.033)	0.624
4	-0.055(0.023)	0.016*	-0.076(0.033)	0.022*	0.018(0.022)	0.418	0.008(0.032)	0.803
5	-0.053(0.022)	0.014*	-0.063(0.031)	0.045*	0.021(0.021)	0.320	0.001(0.030)	0.971
6	-0.050(0.021)	0.015*	-0.051(0.029)	0.082	0.022(0.019)	0.254	-0.004(0.028)	0.882
7	-0.046(0.019)	0.016*	-0.040(0.026)	0.133	0.023(0.018)	0.209	-0.008(0.025)	0.761
8	-0.042(0.018)	0.019*	-0.030(0.023)	0.197	0.022(0.016)	0.180	-0.010(0.023)	0.662
9	-0.038(0.017)	0.022*	-0.023(0.021)	0.268	0.021(0.015)	0.160	-0.011(0.021)	0.583
10	-0.033(0.015)	0.027*	-0.017(0.018)	0.344	0.019(0.013)	0.146	-0.012(0.018)	0.520
11	-0.029(0.014)	0.032*	-0.012(0.015)	0.420	0.017(0.012)	0.136	-0.011(0.016)	0.470
12	-0.025(0.012)	0.038*	-0.009(0.013)	0.495	0.016(0.010)	0.129	-0.011(0.014)	0.429

Significance: $^{*}p < 0.05$, $^{**}p < 0.01$, $^{***}p < 0.001$.

Supplementary Table 4. tDCS effect of block-to-block hierarchy learning in the reaction time of Social condition

	Anode vs Sham				***Cathode vs Sham***			
	**Training**		**Test**		**Training**		**Test**	
Block	**b(SE)**	**P**	**b(SE)**	**P**	**b(SE)**	**P**	**b(SE)**	**P**
1	-32.440(37.704)	0.390	35.783(41.108)	0.384	-27.055(37.704)	0.473	2.289(41.108)	0.956
2	-22.860(37.052)	0.537	45.753(40.126)	0.254	-26.770(37.052)	0.470	3.080(40.126)	0.939
3	-13.280(36.522)	0.716	55.723(39.323)	0.156	-26.485(36.522)	0.468	3.871(39.323)	0.922
4	-3.7008(36.120)	0.918	65.693(38.710)	0.090	-26.200(36.120)	0.468	4.663(38.710)	0.904
5	5.879(35.849)	0.870	75.663(38.296)	0.048*	-25.915(35.849)	0.470	5.454(38.296)	0.887
6	15.459(35.713)	0.665	85.633(38.087)	0.025*	-25.630(35.713)	0.473	6.245(38.087)	0.870
7	25.038(35.713)	0.483	95.604(38.087)	0.012*	-25.345(35.713)	0.478	7.037(38.087)	0.853
8	34.618(35.849)	0.334	105.574(38.296)	0.006**	-25.060(35.849)	0.485	7.828(38.296)	0.838
9	44.198(36.120)	0.221	115.544(38.710)	0.003**	-24.775(36.120)	0.493	8.619(38.710)	0.824
10	53.777(36.522)	0.141	125.514(39.323)	0.001**	-24.491(36.522)	0.502	9.411(39.323)	0.811
11	63.357(37.052)	0.087	135.484(40.126)	0.001**	-24.206(37.052)	0.514	10.202(40.126)	0.799
12	72.937(37.704)	0.053	145.454(41.108)	<.0001 ***	-23.921(37.704)	0.526	10.993(41.108)	0.789

Significance: $^{*}p < 0.05$, $^{**}p < 0.01$, $^{***}p < 0.001$.

Supplementary Table 5. tDCS effect of block-to-block hierarchy learning in the % performance of Non-Social condition

	Anode vs Sham				***Cathode vs Sham***			
	**Training**		**Test**		**Training**		**Test**	
Block	**b(SE)**	**P**	**b(SE)**	**P**	**b(SE)**	**P**	**b(SE)**	**P**
1	0.007(0.026)	0.788	-0.053(0.036)	0.142	0.022(0.026)	0.403	-0.004(0.037)	0.907
2	0.005(0.025)	0.828	-0.045(0.035)	0.197	0.010(0.025)	0.682	-0.017(0.036)	0.639
3	0.004(0.024)	0.873	-0.037(0.034)	0.272	-0.001(0.024)	0.969	-0.027(0.034)	0.422
4	0.002(0.023)	0.920	-0.029(0.032)	0.364	-0.011(0.023)	0.631	-0.036(0.033)	0.268
5	0.001(0.022)	0.966	-0.022(0.031)	0.468	-0.019(0.022)	0.371	-0.043(0.031)	0.168
6	0.000(0.021)	0.992	-0.016(0.029)	0.575	-0.026(0.021)	0.207	-0.048(0.030)	0.107
7	-0.001(0.019)	0.955	-0.011(0.026)	0.680	-0.031(0.020)	0.115	-0.050(0.028)	0.071
8	-0.002(0.018)	0.923	-0.007(0.024)	0.778	-0.034(0.018)	0.066	-0.052(0.026)	0.049*
9	-0.002(0.016)	0.896	-0.004(0.022)	0.867	-0.035(0.017)	0.040*	-0.052(0.025)	0.035*
10	-0.002(0.015)	0.873	-0.001(0.02)	0.947	-0.035(0.016)	0.026*	-0.051(0.023)	0.027*
11	-0.002(0.013)	0.854	0.000(0.017)	0.983	-0.035(0.015)	0.018*	-0.049(0.021)	0.021*
12	-0.002(0.012)	0.838	0.002(0.015)	0.921	-0.033(0.013)	0.013*	-0.046(0.020)	0.018*

Significance: $^{*}p < 0.05$, $^{**}p < 0.01$, $^{***}p < 0.001$.

Supplementary Table 6. tDCS effect of block-to-block hierarchy learning in the reaction time of Non-Social condition

	Anode vs Sham				***Cathode vs Sham***			
	**Training**		**Test**		**Training**		**Test**	
Block	**b(SE)**	**P**	**b(SE)**	**P**	**b(SE)**	**P**	**b(SE)**	**P**
1	-79.414(37.704)	0.035*	-87.487(41.108)	0.033*	-62.531(37.704)	0.097	-93.396(41.108)	0.023*
2	-71.176(37.052)	0.055	-73.214(40.126)	0.068	-53.684(37.052)	0.147	-74.721(40.126)	0.063
3	-62.937(36.522)	0.085	-58.940(39.323)	0.134	-44.837(36.522)	0.220	-56.046(39.323)	0.154
4	-54.698(36.120)	0.130	-44.667(38.710)	0.249	-35.990(36.120)	0.319	-37.371(38.710)	0.334
5	-46.460(35.849)	0.195	-30.394(38.296)	0.427	-27.143(35.849)	0.449	-18.695(38.296)	0.625
6	-38.221(35.713)	0.285	-16.121(38.087)	0.672	-18.296(35.713)	0.608	-0.020(38.087)	1.000
7	-29.982(35.713)	0.401	-1.847(38.087)	0.961	-9.449(35.713)	0.791	18.655(38.087)	0.624
8	-21.744(35.849)	0.544	12.426(38.296)	0.746	-0.602(35.849)	0.987	37.330(38.296)	0.330
9	-13.505(36.120)	0.708	26.699(38.710)	0.490	8.245(36.120)	0.819	56.005(38.71)	0.148
10	-5.267(36.522)	0.885	40.972(39.323)	0.297	17.092(36.522)	0.640	74.680(39.323)	0.058
11	2.972(37.052)	0.936	55.246(40.126)	0.169	25.938(37.052)	0.484	93.355(40.126)	0.020*
12	11.211(37.704)	0.766	69.519(41.108)	0.091	34.785(37.704)	0.356	112.03(41.108)	0.006**

Significance: $^{*}p < 0.05$, $^{**}p < 0.01$, $^{***}p < 0.001$.
