# Supplementary material for: Transcranial direct current stimulation suggests a causal role of the medial prefrontal cortex in learning social hierarchy
Source: Commun Biol. 2024 Mar 9;7:304. doi: 10.1038/s42003-024-05976-2 (PMC10924847; doi:10.1038/s42003-024-05976-2)
Supplement: Supplementary file 4 — Reporting Summary [file 42003_2024_5976_MOESM4_ESM.pdf]

nature portfolio

Corresponding author(s): Jean-Claude Dreher

Last updated by author(s): Feb 12, 2024

Reporting Summary

Nature Portfolio wishes to improve the reproducibility of the work that we publish. This form provides structure for consistency and transparency in reporting. For further information on Nature Portfolio policies, see our Editorial Policies and the Editorial Policy Checklist.

Statistics

For all statistical analyses, confirm that the following items are present in the figure legend, table legend, main text, or Methods section.

n/a	Confirmed	
☐	☒	The exact sample size (*n*) for each experimental group/condition, given as a discrete number and unit of measurement
☐	☒	A statement on whether measurements were taken from distinct samples or whether the same sample was measured repeatedly
☐	☒	The statistical test(s) used AND whether they are one- or two-sided<br>*Only common tests should be described solely by name; describe more complex techniques in the Methods section.*
☒	☐	A description of all covariates tested
☐	☒	A description of any assumptions or corrections, such as tests of normality and adjustment for multiple comparisons
☐	☒	A full description of the statistical parameters including central tendency (e.g. means) or other basic estimates (e.g. regression coefficient) AND variation (e.g. standard deviation) or associated estimates of uncertainty (e.g. confidence intervals)
☐	☒	For null hypothesis testing, the test statistic (e.g. *F*, *t*, *r*) with confidence intervals, effect sizes, degrees of freedom and *P* value noted<br>*Give P values as exact values whenever suitable.*
☒	☐	For Bayesian analysis, information on the choice of priors and Markov chain Monte Carlo settings
☒	☐	For hierarchical and complex designs, identification of the appropriate level for tests and full reporting of outcomes
☐	☒	Estimates of effect sizes (e.g. Cohen's *d*, Pearson's *r*), indicating how they were calculated

Our web collection on statistics for biologists contains articles on many of the points above.

Software and code

Policy information about availability of computer code

Data collection	Using E-prime 2.0 to program the task, presented in 14-inch laptop
Data analysis	STATA 14

For manuscripts utilizing custom algorithms or software that are central to the research but not yet described in published literature, software must be made available to editors and reviewers. We strongly encourage code deposition in a community repository (e.g. GitHub). See the Nature Portfolio guidelines for submitting code & software for further information.

Data

Policy information about availability of data

All manuscripts must include a data availability statement. This statement should provide the following information, where applicable:

- Accession codes, unique identifiers, or web links for publicly available datasets
- A description of any restrictions on data availability
- For clinical datasets or third party data, please ensure that the statement adheres to our policy

The behavioral data of this study are all available as a repository on the Open Science Framework: https://osf.io/zvcfh/.

Human research participants

Policy information about studies involving human research participants and Sex and Gender in Research.

Reporting on sex and gender	We have collected and reported sex in formation in our study. We did not perform analysis on sex difference. Our study was tend to recruit balanced sex number (Female=69, Male=67) to avoid possible sex unbalance influence the results of our study.
Population characteristics	see below (Behavioral & social sciences study design)
Recruitment	Participants were recruited via online fliers with informed written consent. All participants were right-handed, with no history of psychiatric or neurologic disorders
Ethics oversight	the ethics committee of South China Normal University

Note that full information on the approval of the study protocol must also be provided in the manuscript.

Field-specific reporting

Please select the one below that is the best fit for your research. If you are not sure, read the appropriate sections before making your selection.

☐ Life sciences ☒ Behavioural & social sciences ☐ Ecological, evolutionary & environmental sciences

For a reference copy of the document with all sections, see nature.com/documents/nr-reporting-summary-flat.pdf

Behavioural & social sciences study design

All studies must disclose on these points even when the disclosure is negative.

Study description	Non-invasive transcranial direct current brain stimulation (tDCS) study with between-subject double-blind design.
Research sample	N=136 (67 males, 69 females), student sample from south china normal university.
Sampling strategy	Subjects were randomly assigned to sham, anode, or cathode stimulation conditions. The sample size was determined by previous studies involving tDCS stimulation over Prefrontal cortex.
Data collection	behavioral data (computer keyboard responses during an experimental task)
Timing	November 2017 until June 2018
Data exclusions	For the main analysis, 8 participants were excluded. 6 participants did not reach this priori threshold and were excluded from the analysis. One participant was excluded because he responded stereotypically (i.e., one key for the whole block), and another because the program was restarted twice. After the exclusion, the data of 128 participants were maintained for the analysis (Anode=42, Sham=44, Cathode=42).<br>During the analysis of confidence ratings in the test phase, 9 participants were further excluded due to operational issues, which the program did not record their responses. 1 participant was excluded from the analysis because she consistently chose the lowest rating, "1," even though she had a 100% accuracy rate. After the exclusion, the data of 118 participants were maintained for the confidence rating analysis (Anode=39, Sham=38, Cathode=41).
Non-participation	there were no drop-outs
Randomization	Participant were randomly assigned to receive anode, cathode, or sham stimulation over the medial prefrontal cortex (mPFC) while performing the hierarchy learning tasks.

Reporting for specific materials, systems and methods

We require information from authors about some types of materials, experimental systems and methods used in many studies. Here, indicate whether each material, system or method listed is relevant to your study. If you are not sure if a list item applies to your research, read the appropriate section before selecting a response.

Materials & experimental systems

n/a	Involved in the study
☒	☐ Antibodies
☒	☐ Eukaryotic cell lines
☒	☐ Palaeontology and archaeology
☒	☐ Animals and other organisms
☒	☐ Clinical data
☒	☐ Dual use research of concern

Methods

n/a	Involved in the study
☒	☐ ChIP-seq
☒	☐ Flow cytometry
☒	☐ MRI-based neuroimaging
